# Trade-Offs and Constraints in Allosteric Sensing

**DOI:** 10.1371/journal.pcbi.1002261

**Published:** 2011-11-03

**Authors:** Bruno M.C. Martins, Peter S. Swain

**Affiliations:** 1 Centre for Systems Biology at Edinburgh, The University of Edinburgh, Edinburgh United Kingdom; 2 PDBC, Instituto Gulbenkian de Ciência, Oeiras, Portugal; University of Basel, Switzerland

## Abstract

Sensing extracellular changes initiates signal transduction and is the first stage of cellular decision-making. Yet relatively little is known about why one form of sensing biochemistry has been selected over another. To gain insight into this question, we studied the sensing characteristics of one of the biochemically simplest of sensors: the allosteric transcription factor. Such proteins, common in microbes, directly transduce the detection of a sensed molecule to changes in gene regulation. Using the Monod-Wyman-Changeux model, we determined six sensing characteristics – the dynamic range, the Hill number, the intrinsic noise, the information transfer capacity, the static gain, and the mean response time – as a function of the biochemical parameters of individual sensors and of the number of sensors. We found that specifying one characteristic strongly constrains others. For example, a high dynamic range implies a high Hill number and a high capacity, and vice versa. Perhaps surprisingly, these constraints are so strong that most of the space of characteristics is inaccessible given biophysically plausible ranges of parameter values. Within our approximations, we can calculate the probability distribution of the numbers of input molecules that maximizes information transfer and show that a population of one hundred allosteric transcription factors can in principle distinguish between more than four bands of input concentrations. Our results imply that allosteric sensors are unlikely to have been selected for high performance in one sensing characteristic but for a compromise in the performance of many.

## Author Summary

Sensing environmental changes is the first step in the process of cellular decision-making, but many different biochemical sensors exist and why one sensor is selected for a particular task over another is not known. Here we study the sensing properties of a simple and generic allosteric sensor to understand the effectiveness and limitations of its “design”. We begin by defining and calculating a set of six engineering-inspired characteristics of the sensor’s response and investigate how specifying a high performance in one characteristic constrains the sensor’s performance in others. We determine many such trade-offs and, perhaps surprisingly, that much of the space of characteristics is inaccessible given biophysically plausible ranges of parameters. Our results suggest that allosteric sensors are not under selection for high performance in one sensing characteristic but for a compromise in performance between many. Our approach provides both quantitative and qualitative insights about the function and robustness of allosteric sensors and as such is applicable to both the study of endogenous systems and the design of synthetic ones.

## Introduction

Sensing is fundamental to life. All cells detect chemicals in their environment and modify their physiology in response to the chemicals detected. Yet fluctuations in the concentrations of extracellular chemicals and stochasticity in intracellular biochemistry confound the cellular “decision” of which physiological change is most appropriate [1]. Sensing extracellular changes is one of the first stages of such decision-making, but why different biochemical networks use different sensing biochemistry is largely unknown. In eukaryotes, signal transduction in some networks is initiated by, for example, G protein-coupled receptors [2] and by receptor tyrosine kinases [3] in others. Initiation can even occur directly in the case of nuclear receptors [4]. In bacteria, the sensing of extracellular changes can be relayed through two-component signalling systems [5] or directly transduced into changes in gene regulation through allosteric transcription factors [6].

Here we consider the advantages and disadvantages of one of the simplest sensors, common in microbes, the allosteric transcription factor. The activity of allosteric sensors is regulated by their interaction with the molecules they sense. Thinking of information transfer, we will refer to these molecular signals as input molecules (to the sensing system). Such input molecules bind to a site that is distinct from the DNA-binding site of the sensor. Allosteric sensors are often considered to have two main conformations [5], [7], [8], [9], [10], [11] and stabilise into one of these conformations upon binding an input molecule. The stabilized conformation may lead to new gene expression and either favours binding of the sensor to DNA if, for example, the sensor is a transcriptional activator or disfavours DNA-binding if the sensor is a transcriptional repressor.

In general, sensors require several different characteristics to perform well. A sensor should generate outputs that are distinguishable through, for example, having a wide extent of possible outputs (a high dynamic range to use terminology from engineering). For some systems, a sensor ought to respond only to changes in the input that are sufficiently large: the input-output response curve should be sigmoidal rather than hyperbolic. A sensor should not be too “noisy” because changes in the output should be related as best as possible to changes in the input and not be generated by intrinsic fluctuations of the sensing biochemistry if the sensor is to transfer information despite these intrinsic fluctuations. It may also be beneficial if sensors filter any fast dynamics of the input because such changes may be “input noise” and unrelated to the slower extracellular change of interest. A sensor ought to be able to detect small changes in the input by amplifying these changes to large changes in the output: it should have a high gain. Finally, the time taken to sense is important – organisms with precise, but slow sensors may be outcompeted by organisms that respond quickly if not always appropriately – as too is the metabolic cost of synthesizing and maintaining sensors and of the sensing itself.

For any particular biochemical network, it is challenging to know which of these sensing characteristics is favoured. Fast sensing may be important for responding appropriately to an increase in temperature whereas slow but accurate inference of the state of nutrients in the environment may be preferred before initiating sporulation. Using an established model of allosteric transcription factors, we will determine how six sensing characteristics change as the biochemical parameters of individual sensors and the number of sensors alter. Our approach is inspired by that of Detwiler et al. who studied G-protein signalling [12]. A similar methodology has also been applied to small RNAs [13] and to aptamers [14], but we extend the approach by using mutual information to quantify interdependences between the characteristics of a collection of sensors, each sensor having randomly chosen biochemical parameters.

Our goal is to understand biological design. We wish to develop biophysically plausible hypotheses to explain why one sensing system might have, for example, hundreds of allosteric sensors that are dimers and another have tens of sensors that are tetramers. To do so, we should discover which biochemical parameters predominantly determine which sensing characteristic and how the different characteristics “play off” against each other. In engineering, for example, a compromise must be reached between the gain and the bandwidth when designing an amplifier [15]. We will investigate whether analogous “rules-of-thumb” exist for allosteric sensing. From the perspective of synthetic biology, we also wish to know which parameters to manipulate to determine particular sensing properties and whether all regions in the space of characteristics can be reached with biophysically realistic values of parameters.

We begin with the Monod-Wyman-Changeux (MWC) model of allostery [10] and consider six characteristics of allosteric sensing: the dynamic range and the Hill number of the average response, the intrinsic noise, the capacity, the mean static gain and the average response time. We wish to uncover the trade-offs between these characteristics and understand the constraints they pose on allosteric sensing.

## Results

### The MWC model of a sensor

The classic description of an allosteric sensor is the MWC model [10], which forms the basis of our analysis. We assume that sensors can exist in two conformational states, which differ in their quaternary structure and properties of interaction. We will call these states the T state, or the inactive state, and the R state, or the active state. In the absence of any input molecules, the sensor has an intrinsic bias towards the T state.

**Figure 1:**
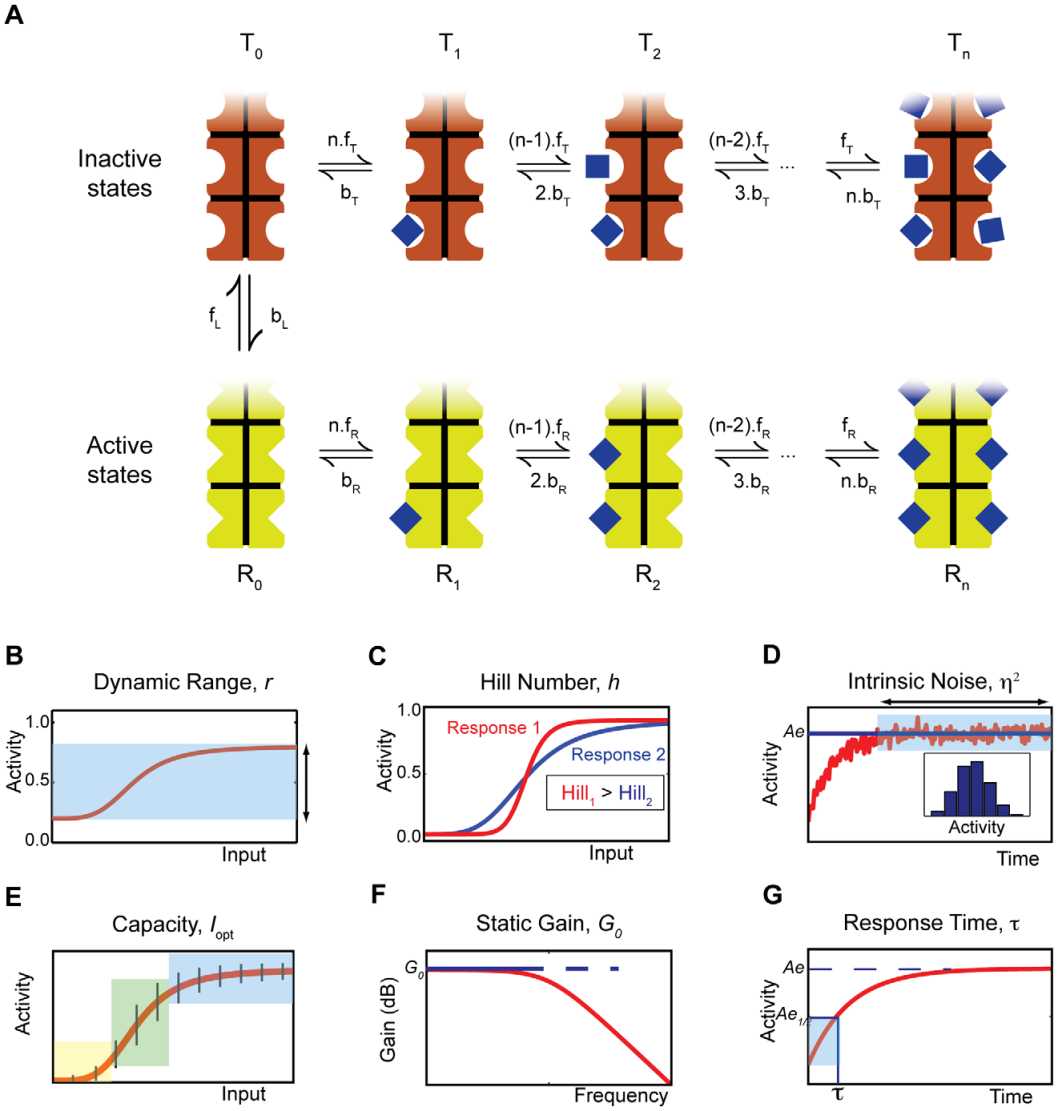
The MWC model of an allosteric sensor and illustrations of the different sensing characteristics of the system. (**A**) The sensor exists in two conformational states, which have different affinities for the signal molecule being sensed. A sensor can transition from one conformation to another only when not bound by a signal molecule, but adding extra transitions does not alter our results. (**B**) The dynamic range, *r*, is the difference between the saturation and basal levels of activity. (**C**) The Hill number, *h*, is a measure of the steepness of the response curve and, indirectly, of the cooperativity of the activation of the sensors and the non-linearity of the response. (**D**) The intrinsic noise, 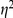, quantifies the relative magnitude of the intrinsic fluctuations in the numbers of active sensors. Inset: histogram of the levels of activity at equilibrium. (**E**) The capacity, *I*_opt_, provides an upper bound on the number of states that can be sensed and distinguished despite intrinsic noise (represented by the black vertical bars). In this example, the sensing system can distinguish between 3 states: low (yellow), medium (green) and high (blue). (**F**) The static gain, *G*_0_, is the change in activity in response to a small step increment in the input signal. The frequency-dependent gain (red curve) decreases as frequency increases: the system is a low-pass filter. (**G**) The response time, 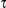, measures the time to reach the level of activity corresponding to half of its equilibrium level.

The sensor detects a signal by binding to input molecules. Such molecules bind preferentially to the R state and so counteract the intrinsic bias towards the T state (Fig. 1A). Consequently, an individual sensor will spend more time in the R state when bound by an input molecule than when unbound, and the equilibrium between R and T states of the population of sensors that are unbound by input molecules will also shift to favour more R-sensors. Both effects encourage the binding of additional input molecules.

We considered sensors that consist of a number of identical subunits, each with its own allosteric binding site. Our analysis, though, applies for any kind of protein with allosteric binding sites that have identical properties when interacting with the same type of input molecule. Following Monod et al. [10], we assumed concerted transitions: all subunits change simultaneously. The active and inactive conformations are therefore properties of the sensor as a whole and not just of the individual subunits. Transitions between the R and T states can occur regardless of how many molecules of the input are already bound to the sensor, but the behaviour of the system is largely unchanged if they are assumed to only occur when the sensor is not bound by input molecules. Transitions between the R and T states when the sensor is bound do not change the concentrations at equilibrium because the product of the kinetic rates in the cycle 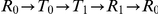 (Fig. 1A) is the same as the product of the rates in the cycle 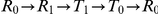 (at equilibrium, the average time taken to go round the cycle should be the same in each direction). Hence, the kinetic rates *f_L_*, *b_L_*, *f_R_*, *b_R_*, *f_T_*, and *b_T_* in Fig. 1A are sufficient to completely determine the concentrations of the various states at equilibrium, even in the presence of additional transitions between the R and T states.

We defined the activity of a population of sensors by the fraction of sensors in the active R state. At equilibrium, the activity, 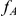, satisfies [10]

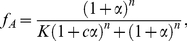
(1)where 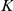 is the allosteric or equilibrium constant of the transition between the R and T states; 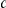 is the ratio of the dissociation constant of the sensor and the input molecule when the sensor is in the R state to the dissociation constant when the sensor is in the T state; 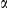 is the concentration of free input molecules in units of the dissociation constant of the R state; and 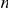 is the number of subunits, or allosteric binding sites, on each sensor. We can think of 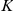 as the bias of a sensor towards the T state in the absence of any input signal and 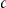 as the counteracting bias towards the R state in the presence of the input. In our analysis, we treat the biases 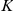 and 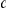 as macroscopic properties of a sensor that do not depend on its number of subunits.

If the number of subunits is greater than one, then, once some sensors in the population have already bound input, the increased probability of additional input molecules binding to the sensors usually generates a sigmoidal response curve, i.e. a non-linear increase in the mean activity for a linear increase in the input. This increase can be sharp and the population of sensors can switch from being mostly inactive to mostly active for a small change in the concentration of input.

### Definitions of the different characteristics of sensing

We used six properties to characterize the reliability and efficiency of sensing:

1) The **dynamic range** (or amplitude), 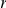, is the difference between the mean basal level of activity, when no input is present, and the mean saturated level, when a high (infinite) concentration of input is present (Fig. 1B). Depending on the strength of the biases 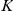 and 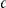 of the sensor, the system can have non-negligible activity in the absence of any input (as high as 50% if 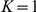) or saturate below the 100% level of activity (saturation will occur at the basal level if 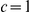). The calculation of the dynamic range gives 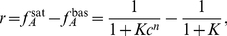
(2)where 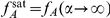 and 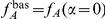.

2) The **Hill number**, 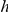, is a measure of the steepness of the switch as the sensors change from being mostly inactive to mostly active, or vice versa, as the concentration of input changes (Fig. 1C). It quantifies the degree of cooperativity of binding of the input molecules. If 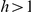, then the binding of one molecule encourages the binding of the next; when 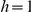, there is no cooperativity and the activity increases hyperbolically with the concentration of input. This characteristic describes, then, the non-linearity of the response and so its ability to generate self-perpetuating dynamics [16], such as bistabilities, through its interactions with downstream components. Mathematically, the Hill number is proportional to the derivative of the response curve at half saturation (in log space). Defining 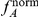 as 
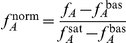
 so that the normalised activity lies between zero and one, we can then write 
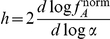
 [17], and so 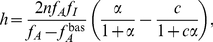
(3)where 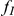 is the fraction of inactive sensors (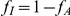) and 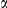 is evaluated at the value of the input, 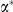, that produces an activity of 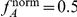: 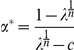
with 
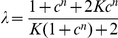
.

3) The **intrinsic noise**, 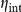, quantifies the relative size of the fluctuations generated by the biochemistry of sensing around the mean level of activity (Fig. 1D). Such fluctuations arise from the stochastic timing of individual chemical reactions. We define 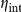 as [18]

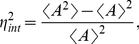
(4)where 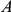 is the number of active sensors,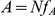, and 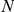 is the total number of sensors in the system. By approximately solving the master equation that describes the MWC model, we found that 
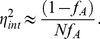
(5)


4) The **capacity**, 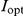, provides an upper bound on the number of levels of input that can be sensed and distinguished given intrinsic noise (Fig. 1E). The capacity is found by maximising the mutual information [19] between the input 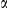 and the activity 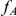. For low levels of intrinsic noise [20], we found that
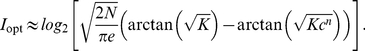
(6)


5) The **static gain**, 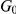, describes the mean change in activity in response to a small step increment in the input (Fig. 1F). The frequency-dependent gain can be found by linearizing an ordinary differential equation model of the system around the equilibrium concentrations (Methods) and takes the general form [15]

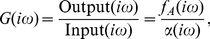
(7)where all quantities are Laplace transformed and 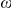 is the angular frequency. Fluctuations in the input represent extrinsic fluctuations. The frequency-dependent gain measures the response of the system to extrinsic variation, and consequently the system’s ability to track small changes in the input. The static gain is defined as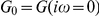 and can be obtained from Eq. (7), but it can also be calculated by differentiating the steady-state activity with respect to the input [12]:
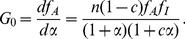
(8)


From the frequency-dependent gain, Eq. (7), we find that the sensing system is a low pass filter (see Methods). It can adapt its activity to slow fluctuations of the input, but gradually loses the ability to respond as the fluctuations become more rapid (Fig. 1F). Beyond a cut-off frequency, the frequency-dependent gain declines, and we find that the response time determines this cut-off frequency, as expected. The filtering properties of the system at high frequencies are independent of the number of allosteric subunits in each sensor: the frequency-dependent gain falls as 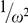 for all *n* (Methods).

6) The **response time**, 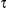, is the time the system takes to reach the level of activity that is equidistant between the basal level and the maximum level for a particular concentration of input. We assume that initially there is a basal level of activity and that input undergoes a step increase from zero (Fig. 1G).

#### Properties of the sensing characteristics

**Figure 2:**
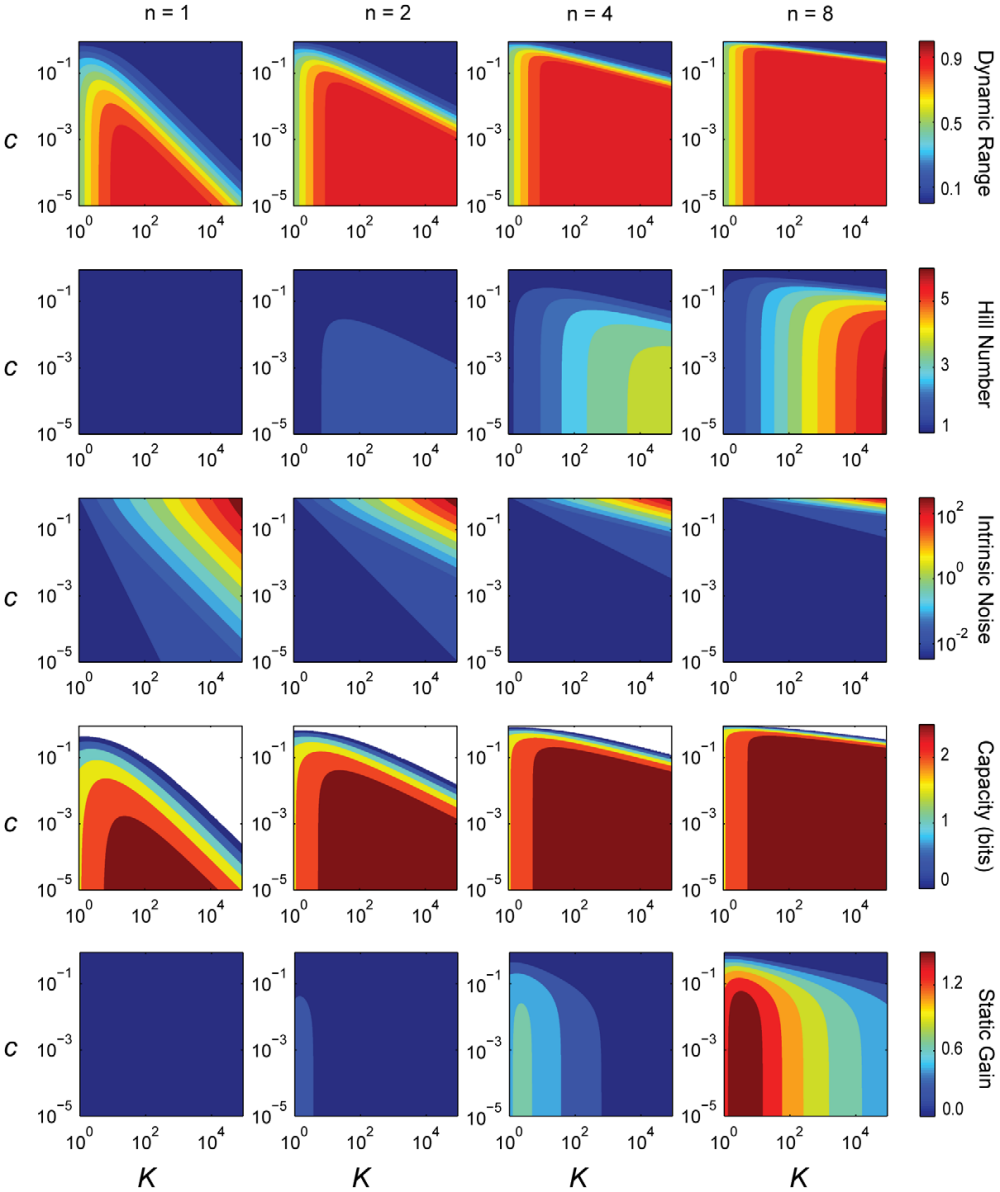
The biases *K* and *c* and the number of subunits *n* completely determine the value of all characteristics except the response time, and consequently each characteristic is not independent. Contour plots of the five characteristics we can derive analytically from Eq. (1). From top to bottom: the dynamic range, the Hill number, the intrinsic noise at the threshold, the capacity, and the static gain at the threshold. From left to right, the number of subunits *n* is respectively 1, 2, 4 and 8. The total number of sensors in the system is 100 and is used to calculate the intrinsic noise and the capacity. The white areas in the contour plots of the capacity (fourth row) correspond to parameter sets for which the magnitude of the intrinsic noise is large enough to invalidate the approximation we use to calculate the capacity. For *n*  = 1, although the Hill number is always one, there is small variation in the static gain (between 0 and 0.06 units).

All characteristics, with the exception of the response time, depend only on the biases 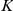 and 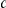, and can be calculated directly, at least for sufficiently high concentrations of input molecules and if the intrinsic noise is low. Fig. 2 shows contour plots of these characteristics for different values of the biases and numbers of subunits *n* and therefore is in some sense the ‘design space’ [21] of the sensors. The intrinsic noise and the static gain are measured at the threshold input: the concentration of input molecules that gives an activity midway between the basal and saturated activities. As expected, we see that the maximum Hill number increases with increasing numbers of subunits and that the capacity is larger when the intrinsic noise is lower. We will focus, however, on five further observations:

1. As the number of subunits increases, the dynamic range becomes restricted to be close to unity for most values of *K* and *c*.
2. A high Hill number implies a high dynamic range.
3. The intrinsic noise at the threshold decreases with increasing numbers of subunits in each sensor.
4. The capacity and the dynamic range are strongly correlated.
5. The static gain and the Hill number are anti-correlated.

To understand their origin, we first consider the system’s intrinsic fluctuations and ability to transfer information.

#### Intrinsic fluctuations

**Figure 3:**
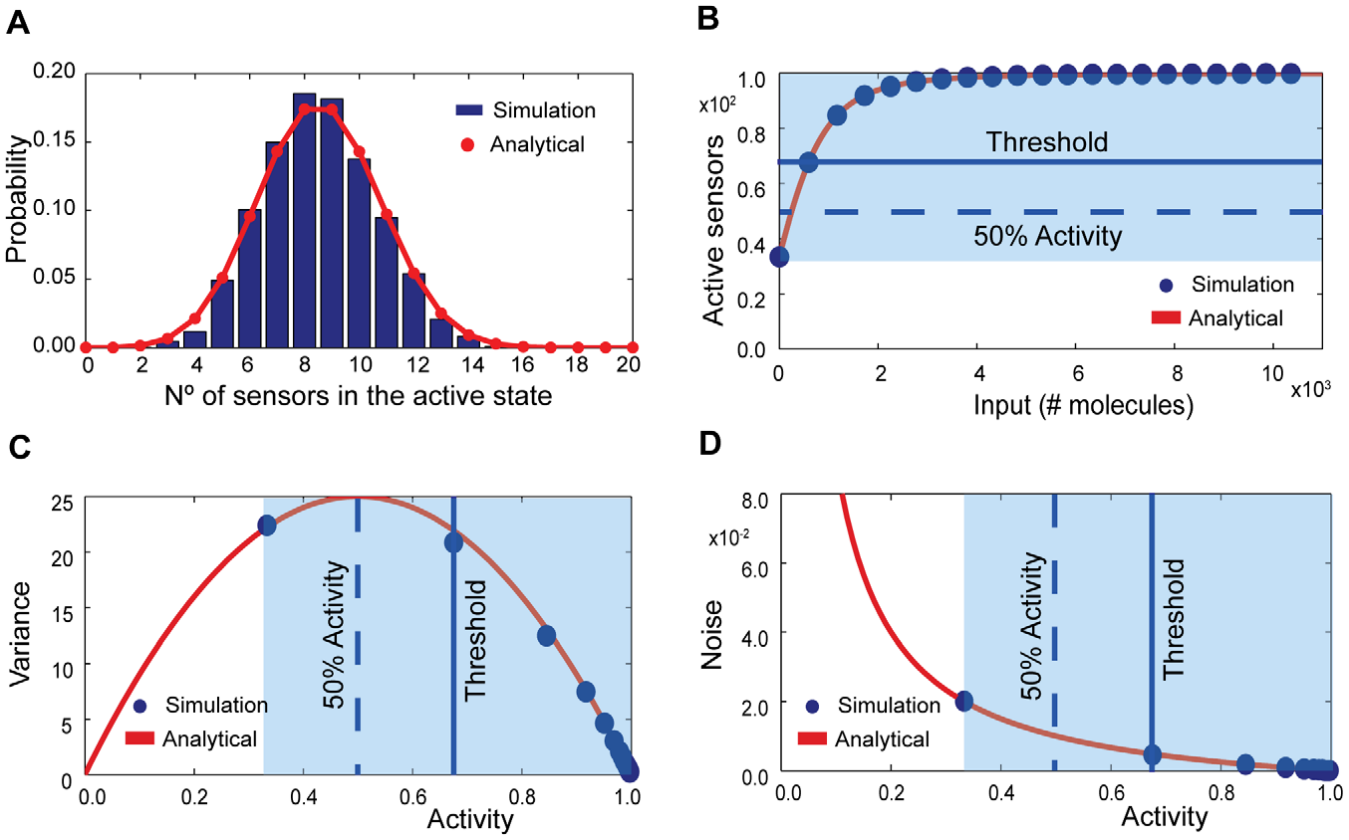
The intrinsic fluctuations follow a binomial distribution and can maximize their variance away from the threshold of the response curve. (**A**) Comparison between Eq. (5) (in dark blue) and numerical simulation (red line). Total number of sensors is 20; the initial number of input molecules is 224; *K* = 100; *c* = 0.01; *n* = 4; *f_R_* =  0.01 s^−1^; *f_T_* =  0.01 s^−1^; *b_R_* =  1 s^−1^; and *b_T_* =  100 s^−1^. (**B**) The threshold of the response (the midpoint between the basal and saturation levels) need not coincide with the level of input at which 50% of the sensors are active. (**C**) The maximum of the variance in the system, which is always located at the 50% of activity level, need not coincide with the threshold value of the input signal. (**D**) The intrinsic noise decreases with increasing input because more sensors become activated. In (**B, C, D**), the dark blue dots represent numerical simulation, the red curve the analytical solutions from Eqs. (1) and (10), and the light blue box shows the dynamic range of activity. The total number of sensors in the system is 100; *K* = 2; *c* = 0.1; *f_R_* = 0.01 s^−1^; *f_T_* = 0.01 s^−1^; *b_R_* = 10 s^−1^; and *b_T_* = 100 s^−1^.

If we assume a concentration of input molecules high enough so that the binding of molecules to the sensors does not substantially reduce the number of free input molecules, then the model of Fig. 1A becomes approximately linear, i.e., all reactions are approximately first order reactions [22]. We can then solve the corresponding master equation (Methods) and find that the probability of having *m* of *N* sensors active approximately obeys a binomial distribution and takes the form
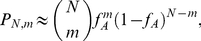
(9)where 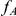 is given by Eq. (1). We verified Eq. (9) using numerical simulations (Fig. 3A).

The mean and variance of the number of active sensors are, respectively,
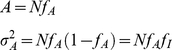
(10)using standard results for a binomial distribution, and where 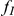 is the fraction of inactive sensors. The variance in the activity is therefore highest when each sensor molecule has exactly a 50% probability of being found in either state and need not occur, as might be expected, at the threshold level of the input. If the system has a high basal level of activity (Fig. 3B), close to 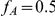, then the highest variance is in the vicinity of the basal level (Fig. 3C). The intrinsic noise given by Eq. (5), however, decreases monotonically with increasing activity because the probability of an individual sensor being active then increases. By raising the basal level of activity sensing systems can thus reduce intrinsic noise at all levels of activity (Fig. 3D).

We note that Eq. (10) implies that the variance in the number of active sensors both determines the Hill coefficient, Eq. (3), and the static gain, Eq. (8), presumably as a consequence of the fluctuation-dissipation theorem [23].

#### Optimizing transmission of information

The capacity is the maximum of the mutual information between the input signal and the output of the system, the activity of the sensors. Mutual information quantifies information transfer [19] and measures how the uncertainty in the input decreases given observations of the output. To calculate the capacity, we must find the optimal distributions of input and output that permit the system to transmit as much information about the input as possible given the system’s intrinsic fluctuations. Following Tkačik et al. [20], we assumed small intrinsic fluctuations and approximate Eq. (9) by a Gaussian distribution. The optimal input distribution, assuming the model of intrinsic noise discussed in the previous section, becomes 
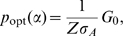
(11)where Z is a normalization constant given by 
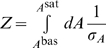
, 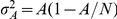 from Eq. (10), and 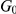 is given by Eq. (8). The optimal output distribution is obtained by dividing the optimal input distribution by the static gain, 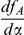, and we find that 
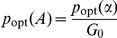
. Evaluating the integral in Eq. (11) and using the definition for mutual information gives Eq. (6) (Methods).

**Figure 4:**
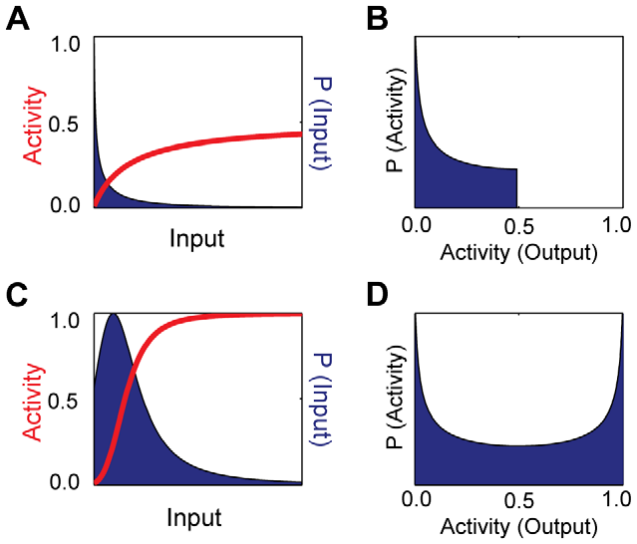
Maximising the information transferred through the sensing system determines optimal distributions for outputs and inputs. (**A**) Optimal distribution of inputs (dark blue area) and activity (red curve) for a system where *n* = 1, *K* = 100, and *c* = 0.01. (**B**) Optimal distribution of outputs for the system in (**A**), which has a dynamic range of about 0.5. (**C**) Optimal distribution of inputs (dark blue area) and activity (red curve) for a system where *n* = 4, *K* = 100, and *c* = 0.01. (**D**) Optimal distribution of outputs for the system in (**C**), whose dynamic range is close to 1.

For optimal information transfer, we find that the input should be distributed mostly where the system is most sensitive and has a steep response curve. Small changes in input will then have changes in output large enough to be distinguishable from changes generated by the system’s intrinsic fluctuations. In Fig. 4A and 4C, we show two examples of the optimal input distributions for sensors with either a hyperbolic or sigmoidal response curve. The most probable values of the input fall where the system is sensitive; the least probable values fall where the system is saturated and has an approximately flat response curve.

The optimal output distribution is bimodal with peaks at the two extremes of activity: 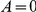 and 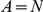 (Fig. 4D). Such a distribution produces distinguishable values of the output. Sensors that have a low dynamic range, however, can only have at most one such peak (Fig. 4B), so they produce only a limited number of distinguishable outputs.

#### The highly sigmoidal limit

A natural and informative limit of the system is when the response becomes highly sigmoidal. This limit requires extreme values of the two biases, 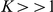 and 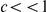, with the combination 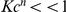. The dynamic range then tends to one with a basal level of zero. The value of input at the threshold grows with *K*: 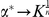. The activity at the threshold tends to a half, and the Hill number tends to its maximum value of *n*. The static gain, however, becomes small with 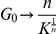.

We now reconsider the observations made from Fig. 2:

#### More subunits increase the dynamic range

For the model of Fig. 1A, increasing the number of subunits on each sensor increases the probability that all sensors can become active for a given concentration of the input: 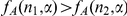 for any 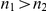 from Eq. (1). The upper limit of the dynamic range in Eq. (2) therefore increases (mathematically, the term 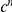 becomes small because *c*<1).

#### A high Hill number implies a high dynamic range

To attain a high Hill number, both biases must be strong with 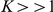 and 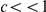, Eq. (3), which is also the condition for a high dynamic range, Eq. (2). Sensing is more cooperative with a sigmoidal response curve of high Hill number and, consequently, we expect that systems with high basal levels or low saturation levels of activity are limited in the degree of cooperativity they can achieve. The sensing response can, however, have relatively low dynamic ranges (∼ 0.7) with Hill numbers of approximately 2, but sensors with eight subunits are required (Fig. 2), which are potentially more expensive to synthesize.

#### The intrinsic noise decreases with the number of subunits

We calculated the intrinsic noise at the threshold level of input, which gives an activity midway between its basal and saturation levels. As the number of allosteric subunits on each sensor increases, the upper limit of the dynamic range grows, and the value of the activity at the threshold level of input also increases. Consequently, more sensors are active, and the intrinsic noise decreases: high intrinsic noise requires low numbers of active sensors (Eq. (5), where 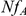 is the number of active sensors, and Fig. 3D).

#### The capacity and the dynamic range are correlated

Intuitively, the larger the dynamic range, the more distinguishable levels of activity are generated for the same range of levels of the input providing there are not competing changes in the magnitude of the intrinsic noise. More information can therefore be transferred. Mathematically, we can see that Eqs. (2) and (6) are both minimal when 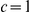 and both maximal when 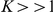 and 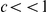. *I*_opt_ can be written as
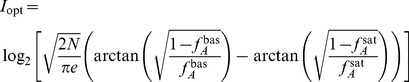
(12)and depends only on the values of the basal and saturated levels of the activity.

To be effective, a sensing system should have a capacity of at least 1 bit and so be able to distinguish between at least two different states of input. For a system with 100 sensors, dynamic ranges of at least 0.5 are necessary to obtain such capacities (Fig. 2). Systems with fewer sensors would have higher intrinsic noise and would therefore require a higher dynamic range.

#### The static gain and the Hill number are anti-correlated

The Hill number is proportional to 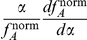: it measures the fractional change in the activity for a fractional change in the input. The static gain, G_0_, is proportional to 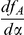: it measures the absolute change in activity for an absolute change in input. Although the Hill number increases with the strength of the biases *K* and *c*, the value of the threshold input also increases, and consequently the slope of the activity curve diminishes. Therefore the static gain decreases. For highly sigmoidal responses, relatively large changes in input are required to generate the expected large change in response because of the increase in the threshold concentration. Small, limiting changes in input generate only small responses. We also note that the Hill coefficient and the static gain are never simultaneously maximal (Fig. 2)

### The space of characteristics has large inaccessible regions

Our results imply that nature is not free to choose each sensing characteristic independently. Specifying certain characteristics restricts the values of others and some regions of characteristic space may even be inaccessible. We used numerical methods to explore generally the effects of one characteristic on another.

We considered the properties of the characteristics for a randomly sampled set of parameter values. We sampled the two biases from a uniform distribution in log-space and so assumed that all orders of magnitude are equally probable. To determine the average response time of the system, we also need the kinetic rates. We sampled these rates similarly to our sampling of the biases and use the values of the biases already sampled to calculate one rate. For example, 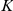 is the ratio of the forward and backward rates in the transition between R_0_ and T_0_ (from Fig. 1A, 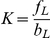 with *f_L_* being the rate of transitioning from R_0_ to T_0_ and *b_L_* being the rate of transitioning from T_0_ to R_0_). Given 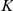 we can then sample freely one of the two rates, but the other is constrained. Similarly, 
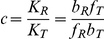
 and constrains another kinetic rate.

Some allosteric sensors may undergo transitions between active and inactive states even when bound by the input ligand, unlike the model of Fig. 1A. Such effects will not change five of the characteristics, but will typically diminish the response time. We therefore compared a model in which only transitions between R_0_ and T_0_ exist to one in which transitions are possible between all states of ligand occupancy. We sample the additional kinetic rates as described above but now constrained to the condition that at each level of occupancy – R_i_ and T_i_ – the ratio of transition rates satisfies 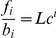. For 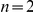, we find that the mean response time is about two orders of magnitude lower when all transitions are considered. Our analysis is not concerned with the absolute values of the response time, but its relation to the other characteristics, and throughout we use the model with transitions only between R_0_ and T_0_ states because, at least for 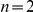, the qualitative behaviour of these relationships is model-independent.

**Figure 5:**
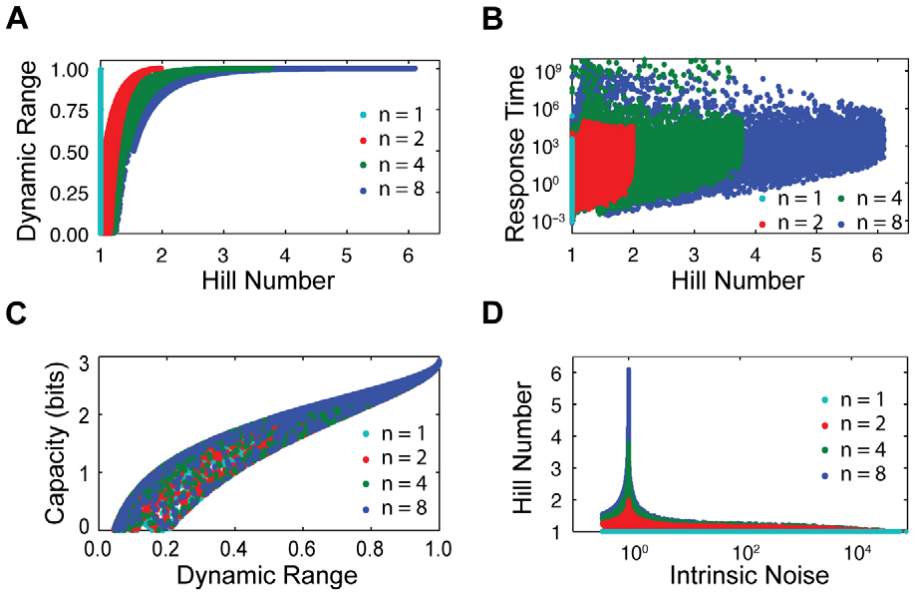
For randomly sampled parameter values, constraints exist between pairs of characteristics. (**A**) Scatterplot of the Hill number and the dynamic range for our randomly sampled parameter sets. (**B**) Scatterplot of the normalised response time (Methods) and the Hill number. (**C**) Scatterplot of the capacity and the dynamic range. (**D**) Scatterplot of the Hill number and the intrinsic noise measured at the threshold of the response curve. For each number of subunits, *n*, there are 10,000 data points.

As well as the general relationships between characteristics we have already discussed, we also observed a weak correlation between the average response time and the Hill number (Fig. 5B). Generating a highly sigmoidal response is biochemically more complex and usually requires more chemical reactions. Consequently, there is a greater probability than one reaction will be slow reducing the overall response time. The correlation is maintained for the 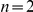 model with all active-inactive transitions but disappears when we only consider simulations where all extra kinetic rates are bound between 10^3^ s^−1^ and 10^−3^ s^−1^ (Methods).

We found that large regions of the space of characteristics are inaccessible. Plotting dynamic range versus Hill number for our randomly sampled parameters (Fig. 5A), we observe a well-defined forbidden region of characteristics space that organisms using allosteric sensing cannot access. There is a tight constraint on the dynamic range when the Hill number is greater than one (Fig. 5A). We can analytically determine the boundary of this region. For example, when 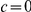 all data points in the characteristic space fall on a line that bounds the points in Fig. 5A from below. Similarly, a scatterplot of capacity and dynamic range (Fig. 5C) shows that the data fall only in a narrow area of characteristic space. Most of the space is inaccessible.

Further, high Hill numbers constrain the intrinsic noise to a narrow band of possible values (Fig. 5D). Attempts at reducing intrinsic noise below this constrained band require sharply reducing the Hill number (for all sensors with more than one subunit). As we have seen, high Hill numbers imply high dynamic ranges (Fig. 5A) with basal levels close to zero and saturation levels close to one and, consequently, the threshold of the response curve will coincide with 50% of the sensors being active. From Eq. (5), the intrinsic noise is then fixed at 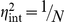, where 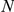 is the number of sensors, and is insensitive to the values of the two biases. Hence the narrow peak observed in Fig. 5D.

When looking at the six-dimensional space of all characteristics, we found most of the space is empty and is inaccessible to allosteric sensing systems: for all numbers of subunits, we find 90% of all sampled systems are contained within less than 2% of the space of characteristics, and all systems lie within less than 6% of the space (in contrast, 10,000 samples of six randomly distributed characteristics would with this measure occupy 100% of the six-dimensional space). The densest regions have relatively high dynamic range and capacity, low intrinsic noise, and relatively low static gain for all numbers of subunits. As the number of subunits increases, both the static gain and the Hill number are more evenly distributed across the densest regions (Fig. 2), and the response time takes average values.

### The sensing characteristics are mutually constraining

**Figure 6:**
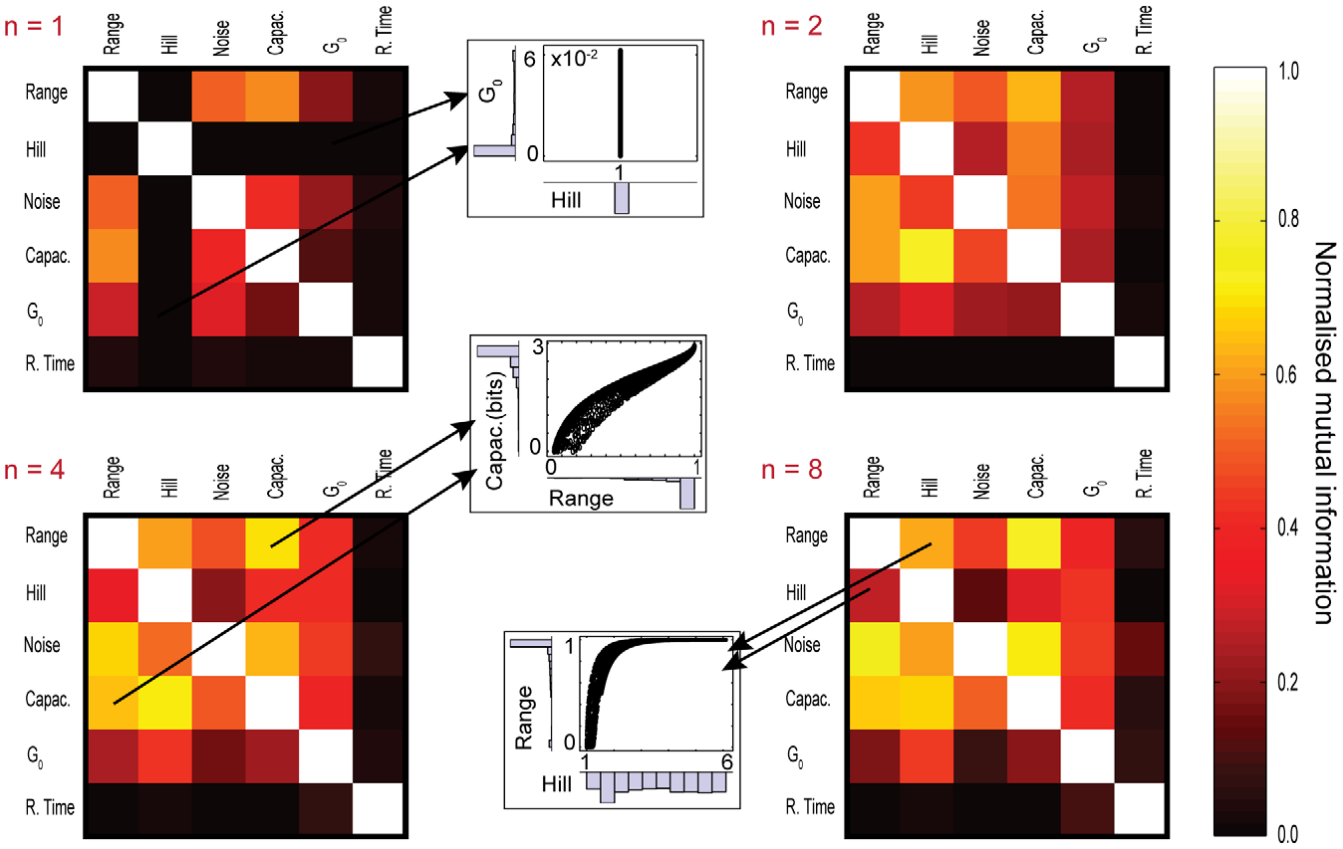
The mutual information between pairs of characteristics quantifies the dependency of one characteristic on another. The four matrices show the mutual information between all pairs of characteristics for different numbers of allosteric binding sites per sensor *n* = 1, 2, 4 and 8. The mutual information is normalised by the entropy of the characteristics on the rows. The darker colours represent pairs that are relatively unconstrained and the brighter colours indicate pairs that are more constrained. The three scatter plots give three examples of different constraints. From top to bottom, we have scatterplots of the static gain versus the Hill number, of the capacity versus the dynamic range, and of the dynamic range versus the Hill number. When 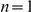, we observe a dark row corresponding to pairs involving the Hill coefficient because the Hill coefficient is always one when 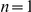. The diagonals are white because the normalised mutual information of a characteristic with itself is always maximal.

To quantify further the trade-offs between pairs of characteristics, we calculated the normalised mutual information [19] between all possible pairs for our randomly sampled parameters (Methods). We normalised by either the entropy of the first or the second characteristic of the pair (Fig. 6). For example, if we normalise by the entropy of the first characteristic, the normalised mutual information measures the fraction of the entropy of the first characteristic that is constrained by specifying the second. When one characteristic is fixed, such as the Hill number for 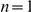, then it shares no mutual information with others (inset of Fig. 6), and changing any other characteristic cannot alter the Hill number. There is a trend for the constraints between characteristics to increase slightly as number of subunits of the sensors increase (compare the colour of the matrix for 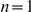 with the matrix for 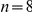 in Fig. 6). The characteristics are therefore more independent for lower numbers of subunits.

The normalised mutual information need not be symmetric: specifying one characteristic can therefore constrain another more than specifying the second characteristic constrains the first. In the 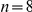 matrix, we emphasize the trade-off between the Hill number and the dynamic range. The entropy of the Hill number is higher than that the entropy of the dynamic range (inset of Fig. 6). Hence specifying the Hill number constrains the dynamic range more than specifying the dynamic range constrains the Hill number. A similar phenomenon occurs for the constraints between the dynamic range and the intrinsic noise (Fig. 6).

We compared the results of the normalised mutual information involving the response time for both the model of Fig.1A and the alternative model with transitions between all active and inactive states when 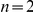. For our sample of simulations the maximal deviation observed between both models was about 70%. The normalised mutual information between the response time and any other characteristic, however, remained low and qualitatively Fig. 6 is unchanged.

## Discussion

For allosteric transcription factors, one of the simplest biological sensing systems, we found several relationships between the system’s sensing characteristics and that specifying one characteristic strongly constrains others (Figs. 2, 5 and 6). Using the Monod-Wyman-Changeux model, we showed that the dynamic range of a collection of sensors reaches its maximum for most values of the two biases, *K* and *c*, particularly as the number of subunits comprising each sensor increases. We found that the Hill number of the mean input-output response curve and the capacity – its ability to transmit outputs that distinguish changes in the system’s input – are both strongly correlated with the dynamic range and that the Hill number is inversely correlated with the static gain. Further, we showed that the intrinsic noise typically decreases as the number of subunits on each sensor increases.

Perhaps surprisingly, we discovered that most of the space of characteristics is inaccessible for typical values of biochemical parameters (Fig. 5). For the collection of parameter values we considered (10,000 sets of parameters in all), constraints between characteristics caused less than 6% of the space of characteristics to be occupied. A sensing system must therefore trade high performance in one characteristic for low performance in another. For example, the intrinsic noise in the response is highest when the number of active sensors is low and therefore will be reduced by increasing the system’s basal level of activity (Fig. 3D). Such an increase, however, diminishes the dynamic range and also therefore the system’s capacity (Fig. 2 and Fig. 5C). The fall in intrinsic noise is not enough to overcome the decrease in capacity caused by reducing the dynamic range. Reducing the noise would also decrease the Hill number (Fig. 5D). The response time, however, would be expected to become faster (Fig. 5B). Such constraints tighten as the number of subunits in each sensor increases (Fig. 6).

To maximise information transfer, the system should generate discriminative outputs for as many bands of input concentrations as possible. We found that the distribution of output that maximized the mutual information between the input and the output is indeed discriminatory being peaked only at low and high values. Similarly, the corresponding optimal input distribution has high probabilities for those inputs where a small change in input gives a large change in the mean output activity and has low probabilities for inputs that give little mean change in the output (Fig. 4). We find that the capacity has a maximum value of around two bits for a system with a population of 100 sensors (Fig. 2), and so allosteric transcription factors can therefore distinguish between four bands of input concentrations, at least when our assumptions of low intrinsic noise and more input molecules than sensors hold. Selection, though, need not favour the ability to distinguish multiple bands and therefore, say, multiple states of the extracellular environment, but rather the ability to quickly and reliably determine a few states, such as the presence or absence of a toxin. Further, some cellular information-processing is likely to be dynamic [24] with the system not having time to reach steady-state as we have assumed here.

The number of subunits a sensor has enables the sensing system to access different regions of the space of characteristics (Fig. 2). Comparing 33 allosteric transcription factors in *Escherichia coli* [25], we found that 33% are monomers and 48% are dimers with only 18% having more than two subunits (Table 1). Being a dimer helps a transcription factor recognise palindromic sequences in promoters [6], but having two subunits also perhaps gives a profitable compromise between the fragility and robustness of the sensing characteristics [26], [27]. Dimeric systems have both substantial regions of parameter space where some characteristics, such as the dynamic range, intrinsic noise, and capacity, vary and equally substantial regions where the dynamic range and the capacity are large and the intrinsic noise small (Fig. 2). Sensors with four or eight subunits, however, will have a near maximal capacity for most values of the two biases, at least when there are 100 sensors and given our approximations (Fig. 2), but do not appear common in *Escherichia coli*. To include the higher biochemical costs of synthesizing sensors with four or eight subunits, we can compare the capacity for equal numbers of subunits rather than equal numbers of sensors (e.g., a system with 50 dimers versus a system with 25 tetramers). From Eq. (6), however, the maximal capacity declines with the total number of sensors favouring dimers over tetramers when the total number of subunits is limiting.

**Table 1:** Numbers of allosteric transcription factors in *E. coli* sorted according to their number of subunits.

Allosteric transcription factors in *Escherichia coli*	
Type of oligomer	Numbers present
Monomers	11
Dimers	16
Tetramers	3
Hexamers	2
Octamers	1

For each of the transcription factors listed in Wall et al. [25], we searched in online databases [42] and in the literature for information on the type of oligomer they form. We excluded those for which no information could be found or for which there are contradicting reports.

An important caveat to our results is that natural selection presumably acts on the entire biochemical system, not only on upstream allosteric sensing but also on both the direct and indirect downstream gene expression. Our analysis, however, is perhaps best extended to specific systems because to understand trade-offs and constraints in those systems we need to know the biochemical details of their control and regulation, the probability distribution of typical inputs, the costs and benefits of potential responses, and ideally how these responses correlate with fitness [1], [28]. Nevertheless, we can make a few general predictions about how some individual characteristics will change if the output of the system is taken to be the level of expression of a downstream gene. For example, the maximum Hill number describing the response of the last species of a biochemical cascade is given by the product of the Hill number at each stage of the cascade [29]. We can expect the capacity to at best remain unchanged with the addition of each new stage in the cascade from the data-processing inequality [19]. Intrinsic noise in the output can increase because each stage of the cascade is itself a new source of stochasticity [30], [31], but need not do so if the input at each stage saturates the output at that stage [32]. Further, assuming that regulation of the downstream gene can be described by a Hill function, we can show by simulation that the dynamic range of the expressed protein is more sensitive to changes in this function’s threshold rather than its Hill number.

If we consider that natural selection acts to improve the performance of a sensing system, our results indicate that the performance of allosteric sensors is likely to be a function that balances the values of all the sensing characteristics. Changing one or two necessarily changes others. Indeed, there are other factors, such as structural constraints, difficulties in regulating the molecular assembly of large oligomers, fluctuations in the number of sensors, and energetic costs, that we have not considered and that will impact selection. Nevertheless, the biochemistry of allosteric sensing prevents random changes in the values of biochemical parameters generating random changes in sensing, and, as such, the constraints we have determined here may themselves have been selected to enable allosteric sensors to be evolvable and reduce catastrophic mutations [33].

## Methods

### Sampling of parameters

The biases 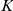 and 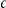 are sampled from a uniform distribution in logarithmic space across six orders of magnitude for each case, i.e., 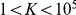 and 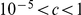. We consider the number of subunits on each sensor to be 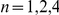 and 8. For each, we sample 10,000 different sets of 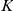 and 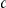 pairs. The kinetic rates 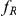, 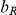, 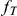 and 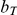 (Fig. 1A) are sampled *a posteriori* with the constraint that the previously sampled values of the biases are maintained. Taking the typical volume of an *E. coli* cell, 10^-18^ m^3^ [34], and the diffusion-limited upper bound on association rates, which is in the order of 10^7^ – 10^10^ M^−1^ s^−1^ [35], we sample normalised kinetic rates between 10^−3^ s^−1^ and 10^3^ s^−1^. We sample the kinetic rates for the model that includes transitions between all active and inactive states similarly, maintaining that 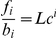.

### Estimating the response time

We find the response time by simulations of an ordinary differential equation model using the *Facile* software [36] and MATLAB (The MathWorks, Massachusetts). We normalise the response time by dividing by 1s, which is the timescale associated with the central value of the range we chose for the kinetic rates.

### Intrinsic noise

We can calculate the intrinsic noise of the system analytically if we assume that the number of input molecules is much greater than the number of the sensors. We can then consider each sensor to act independently: any input molecule binding to one sensor does not affect the availability of input molecules for other sensors because the number of free input molecules is always assumed to be much greater than the number of sensors proteins, or more exactly the total number of allosteric binding sites. We proceed by considering a single allosteric molecule. In the presence of input molecules, the sensor will transition between R and T states according to the transition probabilities for each trans x transitioning from a state to any of its neighbours and the transition probabilities are independent of how the system reached that state. Denoting the time-dependent state vector of the system by 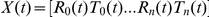 and the transition rate matrix by 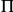, where 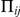 is the transition probability between states i and j and 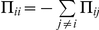, then 
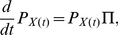
(13)and we have
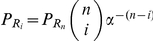
 and 
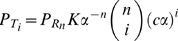
 at steady-state. Eq. (13) is identical to the system of deterministic rate equations that describe the dynamics of the mean activity for a fixed amount of free input. The probability of a sensor being in the active state is given by
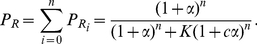
(14)


These results then show the probability of an individual sensor being active is given by 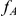 from Eq. (1) – the deterministic (mean) activity for a population of sensors. Furthermore, in a model that includes transitions between the R and T states when they are bound by input molecules, neither the mean activity (Eq. (1)) nor the probability of a sensor being active (Eq. (14)) are altered because of the thermodynamic constraint between parameter values created by the presence of cycles of reactions (involving 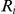, 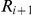, 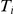 and 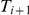).

To extend our results to a stochastic population of sensors, we need to consider all possible configurations of the individual sensors that correspond to a particular activity of the population. For example, for a system with two sensors and one subunit,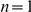, our approximation implies that both sensors are independent and the probability that both are active is then



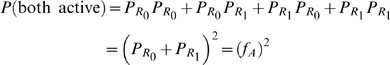
(15)


Similarly, the probability that only one sensor is active is 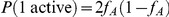 and the probability that no single sensor is active is 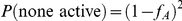. Extending the argument for systems with higher numbers of sensors, the activity obeys a binomial distribution [22]: the probability of having *m* of *N* sensors active is



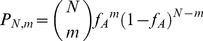
(16)which corresponds to the probability of an activity of *m/N*.

### Calculation of the capacity

The capacity is the upper bound of the mutual information between the input 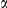 and output, i.e., the activity 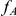, given a model for the intrinsic noise. The mutual information is defined as [19]

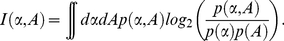
(17)


Using a small Gaussian noise approximation, Tkačik et al. [20] derived the optimal solutions of the input and output that maximise the mutual information, i.e., the capacity. Their solution is 
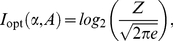
(18)where 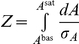. Using the expressions for 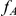 and 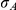 in Eqs. (1) and (9), we find the capacity in the MWC system satisfies Eq. (6).

### Stochastic simulations

All stochastic simulations were performed using the *EasyStoch* software [38], which implements the Gibson-Brück [39] version of the Gillespie algorithm [40].

### Calculation of the frequency-dependent gain

Given the general form of a linear system, 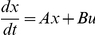, 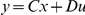, where 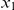, 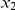,…, 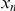 are the state variables, 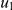, 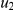,…, 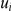 is the input and 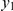, 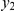,…, 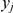 is the output, it can be shown that the frequency response is given by 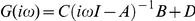 with *I* being the identity matrix [15]. The frequency-dependent gain measures the relation between the input and the output of linear systems, and here we use it to quantify how the system responds to extrinsic fluctuations in the input signal. We ignore intrinsic fluctuations and consider the system to be at equilibrium, or at an ‘operating point’, and introduce a small perturbation term to the input, *L*, so that it becomes 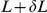. This fluctuation propagates through the system and each variable gains a small correction whose dynamics we follow by linearizing an ordinary differential equation model of the system. We can then write down the matrices 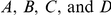
and calculate the frequency-dependent gain directly.

### The sensing system is a low-pass filter

The frequency-dependent gain has the form
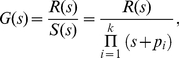
(19)where 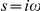 is the complex argument of the Laplace transform. The roots of the numerator,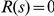, are the zeros of the system; those in the denominator, written as 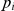, are the poles. The exact number of zeros and poles varies with the number of subunits on the sensors, and the magnitude of the gain rises for each zero and falls for each pole [15]. We found the number of poles always to be greater than the number of zeros and therefore the system is a low-pass filter. We consider the pole with the lowest frequency to be the cut-off frequency. For all numbers of subunits the number of poles exceeds the number of zeros by two, so in the limit of large frequencies the transfer function declines with 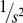.

### Occupancy in the space of characteristics

To give an estimate of the density and occupancy of the space of characteristics, we divided the space of characteristics into equally sized hypercubes and counted the number of sampled sets that fall into each hypercube. We binned each characteristic into low, medium and high levels, thus obtaining a total of 729 hypercubes.

### Quantifying constraints by mutual information

Mutual information is a statistical measure that quantifies how much knowing one random variable informs on another [19], [41]. It is symmetric, but we normalise it in two ways, by the entropy of each characteristic in the pair. For characteristics 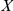 and 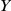, the normalised mutual information in its discretized form is 
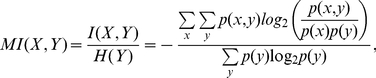
(20)where 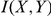 is the mutual information between two characteristics and 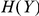 is the entropy of one characteristic. We estimate the probability distributions for the characteristics by binning values of the characteristics from our randomly sampled parameter sets into 30 bins. Although the probabilities are then dependent on the numbers of bins, we varied that number without observing a substantial qualitative change in the mutual information.
